# Evaluation of Zn: WO_3_ Thin Films as a Sensing Layer for Detection of NH_3_ Gas

**DOI:** 10.3390/mi14040732

**Published:** 2023-03-25

**Authors:** Anusha, Priyanka Kumari, P. Poornesh, Saikat Chattopadhyay, Ashok Rao, Suresh D. Kulkarni

**Affiliations:** 1 Department of Physics, Manipal Institute of Technology, Manipal Academy of Higher Education, Manipal 576104, India; 2 Department of Chemistry, School of Basic Sciences, Manipal University Jaipur, Jaipur 303007, India; 3 Department of Physics, School of Basic Sciences, Manipal University Jaipur, Jaipur 303007, India; 4 Department of Atomic and Molecular Physics, Manipal Academy of Higher Education, Manipal 576104, India

**Keywords:** WO_3_, NH_3_ sensing, Zn doping, defects

## Abstract

Pristine WO_3_ and Zn-doped WO_3_ were synthesized using the spray pyrolysis technique to detect ammonia gas. The prominent orientation of the crystallites along the (200) plane was evident from X-ray diffraction (XRD) studies. Scanning Electron Microscope (SEM) morphology indicated well-defined grains upon Zn doping with a smaller grain size of 62 nm for Zn-doped WO_3_ (Zn: WO_3_) film. The photoluminescence (PL) emission at different wavelengths was assigned to defects such as oxygen vacancies, interstitial oxygens, localized defects, etc. X-ray Photoelectron spectroscopy (XPS) studies confirmed the formation of oxygen vacancies in the deposited films. The ammonia (NH_3_) sensing analysis of the deposited films was carried out at an optimum working temperature of 250 °C. The sensor performance of Zn: WO_3_ was enhanced compared to pristine WO_3_ at 1 ppm NH_3_ concentration, elucidating the possibility of the films in sensing applications.

## 1. Introduction

Over the past few decades, chemiresistive-based gas sensors have drawn much attention to the development of the internet of things (IoT) due to the features such as low cost, miniaturization, and low power consumption [1,2,3,4,5]. Different semiconducting oxide materials such as ZnO, WO_3_, SnO_2_, TiO_2,_ etc., were utilized to detect toxic gases such as NO_2_, CO, NH_3_, H_2,_ etc. [6,7,8,9,10,11,12,13]. These gases in the human environment affect physical health by causing headaches, nausea, damage to the central nervous system and severe illness [14]. Among all metal oxides, Tungsten Oxide (WO_3_), a transition group metal oxide, was widely used to detect gases such as NH_3_, NO_2,_ and alcohol vapors [15,16,17]. Ammonia (NH_3_), a reducing gas, is one of the harmful gases produced by chemical plants, automobiles, and agricultural wastes. According to the OSHA report, exposure to ammonia more than the permissible limit (27 mg/m^3^ or 35 ppm for 15 min duration) will severely damage human organs and lead to burns, chemical pneumonitis, and sometimes death [18]. Therefore, it is necessary to have fast and real-time ammonia sensors in order to reduce accidental threats. Furthermore, in the medical field, the lowest detection limit (50 ppb–2 ppm) is required for breath analysis. So, the detection of ammonia is indeed essential in monitoring health and the environment. Recently, WO_3_/Fe_2_O_3_ composites synthesized by the hydrothermal method showed an excellent response to ammonia at a working temperature of 300 °C [19]. Furthermore, a response of 13.6 was reported for Pt-loaded WO_3_ for 200 ppm ammonia [20]. Undoped films hinder sensing performance due to poor sensitivity and selectivity issues [21,22,23]. Hence, metal doping or composite preparation is a crucial step in the synthesis process and in ordering sensing properties.

In this work, we doped WO_3_ with metal dopant Zn due to its comparable cationic radii (0.6 Å for W^6+^; 0.74 Å for Zn^2+^), anticipating the substitution of Zn in the host lattice modulates optical, structural, and electrical properties [24,25]. Additionally, modification of these properties would enhance the gas sensing characteristics of Zn-doped WO_3_ (Zn: WO_3_). B. Nam and co-workers demonstrated the ZnO-WO_3_ composite for CO and NO_2_ sensing at 300 °C and 200 ppm gas concentration [26]. Based on their research, they concluded that the sensor worked better for reducing gas (CO) rather than oxidizing gas (NO_2_). To date, reports on Zn: WO_3_ for ammonia sensing has not been found in the literature. Due to the high operating temperature and high detection limit of the gases, as seen in the above literature, a practical approach to various applications is difficult. Hence, in our present work, we explored the sensing studies of spray-deposited Zn: WO_3_ films towards ammonia up to 1 ppm in concentration and achieved enhancement in the response value at an optimum working temperature of 250 °C.

## 2. Experimental

### 2.1. Chemicals

Ammonium metatungstate hydrate [(NH_4_)_6_H_2_W_12_O_40_·xH_2_O] from Sigma-Aldrich, St. Louis, Mo, USA, Zinc acetate dihydrate [Zn(CH_3_COO)_2_·2H_2_O] from Merck Life Science Pvt. Ltd., Mumbai, India, Polyethylene Glycol 400 (PEG 400) from Molychem, Mumbai, India was used without further purification.

### 2.2. Synthesis and Material Characterization

Pristine WO_3_ and Zn: WO_3_ film at 5 wt.% were synthesized using the spray pyrolysis deposition technique. The desired amount of tungsten and zinc precursors were dissolved in double distilled water to obtain a homogeneous solution of 0.01 M concentration. Solvent PEG 400, which acts as a surfactant, was added to the final solution and stirred using a magnetic stirrer for about 30 min. During deposition, spray parameters such as the flow rate (2 mL/min), the distance from the nozzle to the substrate (19 cm), and substrate temperature (400 °C) were kept constant throughout the experiment.

Structural information was obtained from an X-ray diffractometer that used Cu K-α radiation (1.54 Å). Morphological properties were analyzed via Scanning Electron Microscope. Various defects and sources of origin were determined using a Photoluminescence spectrofluorometer. Structural conformation and vibrational mode information were obtained from the Raman spectrometer. Composition and oxidation state analysis was carried out using X-ray Photoelectron Spectroscope.

### 2.3. Gas sensing Measurements

Synthesized films were kept in a closed chamber and were exposed to the air (79% N_2_ + 21% O_2_) and ammonia gas at different ppm levels. The ammonia concentration was controlled via a flow-through method wherein the carrier gas (air) was mixed with the target gas (NH_3_) with the help of mass flow controllers. I-V measurements were performed using silver paste as electrical contacts and variation in the resistance was noted from the Keithley source meter. The obtained values were used to calculate additional sensing parameters such as sensor response, response, and recovery times.

## 3. Results and Discussion

### 3.1. XRD Studies

**Figure 1:**
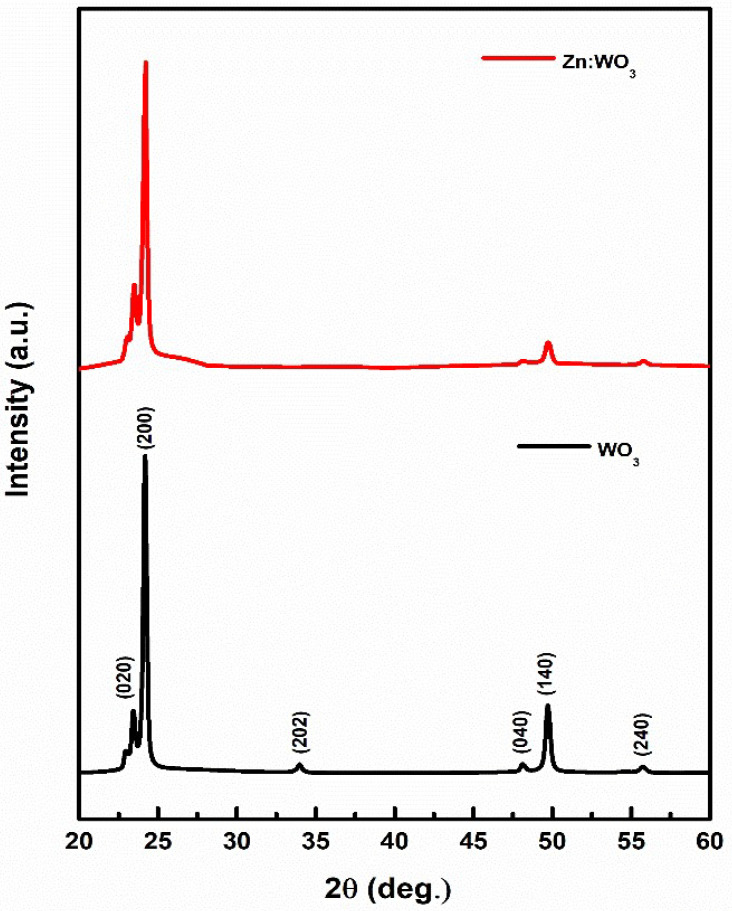
XRD patterns of WO_3_ and Zn: WO_3_ films.

XRD patterns of pristine WO_3_ and Zn: WO_3_ films by the Rigaku SmartLab X-ray diffractometer are illustrated in Figure 1. The diffraction peaks obtained were consistent with the monoclinic structure of WO_3_ and agreed with the JCPDS data card (no. 43-1035). Observed diffraction patterns at 22.97°, 23.44°, 24.18°, 33.99°, 48.15°, 49.73° and 55.74° corresponded to the peaks (002), (020), (200), (202), (040), (140) and (240). It indicates that the films have well crystalline and exhibit a strong preferential orientation (200), which is confirmed by an intense high peak located around 24.18° for both samples. It also confirms an increase in FWHM, which suggests the possibility of lowering the crystallite size, generating a non-uniform strain, or loss in crystalline nature with doping. Again, there was no evidence of any secondary phases corresponding to ZnO or ZnWO_4,_ etc., during doping which validated the pure nature of the deposited samples. A decrease in crystallinity with Zn incorporation was reconfirmed by the reduction in peak intensities with the doping amount. The ionic radius of Zn^2+^ was 0.74 Å, and W^6+^ was 0.60 Å [24]. It is expected that during doping, some Zn^2+^ displace W^6+^ without changing its monoclinic crystal structure. Still, due to size and charge imbalance, a distortion in the crystal structure is reflected in its interplanar spacing values. This may be attributed to microstrain generation in the film during doping. The crystallite size for the dominant peak was estimated via Sherrer’s equation [27] and was found to decrease for the Zn: WO_3_ film. A similar trend was noticed by M.Arshad et al. [28] for Zn-doped WO_3_ nanoparticles. Structural parameters extracted from XRD patterns are given in Table 1.

### 3.2. Raman Studies

**Figure 2:**
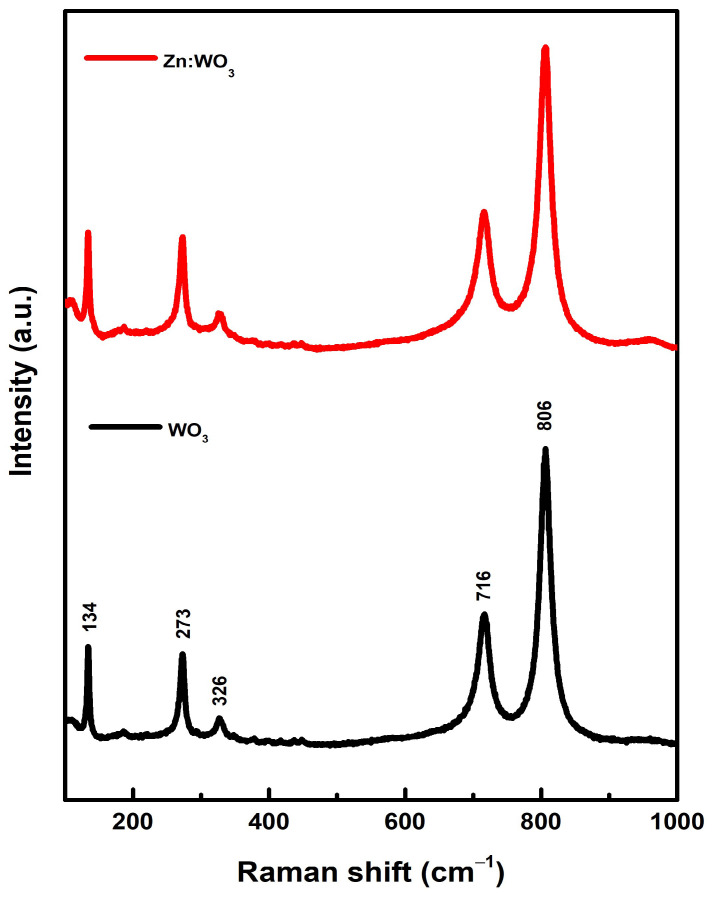
Raman spectra of WO_3_ and Zn: WO_3_ films.

Raman spectra give an insight into crystallinity and bonding within the nanomaterials under investigation. The spectra (Figure 2) were obtained for pristine WO_3_ and Zn: WO_3_ films in 100–1000 cm^−1^ using a Horiba JOBINYVON LabRAM HR spectrometer. Intense wavenumber bands at 716 cm^−1^ and 806 cm^−1^ provided information about O-W-O asymmetric and symmetric stretches. In comparison, bands at 273 cm^−1^ and 326 cm^−1^ corresponded to O-W-O bending vibrations, and these two sets of bands validate the monoclinic phase of crystalline WO_3_ [29,30]. A slight shift (~2 cm^−1^) for bending vibrations might be due to the lattice distortion caused by Zn doping, as Zn^2+^ and W^6+^ have different ionic radii. Bands below 200 cm^−1^ may be attributed due to lattice vibrations. The evolution of a fragile band at 958 cm^1^ for Zn: WO_3_ film corresponds to the symmetric stretching of W=O terminal bonds at the grain surface [31].

### 3.3. SEM Studies

**Figure 3:**
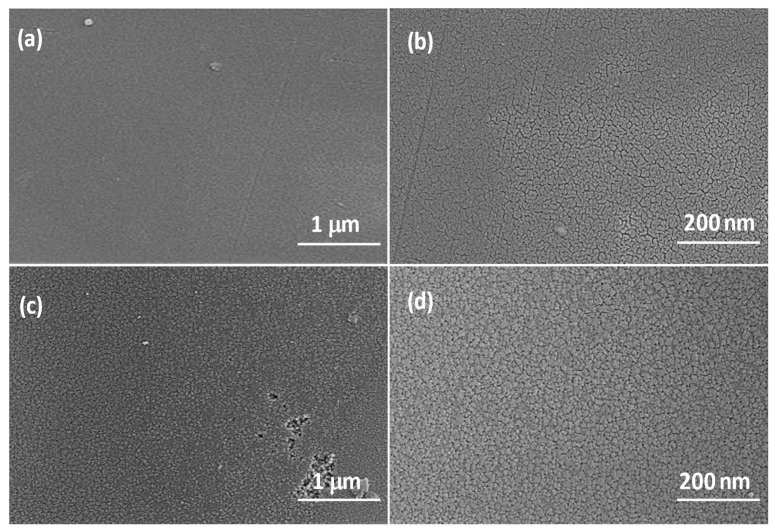
SEM micrographs of (**a**,**b**) WO_3_ and (**c**,**d**) Zn: WO_3_ films.

Morphological features of pristine WO_3_ and Zn: WO_3_ films were investigated via ZEISS ULTRA55 Scanning Electron Microscope. Figure 3a–d illustrates the closely packed grains with uniform distribution all over the surface. It is evident from the obtained morphographs that the grain size of Zn: WO_3_ films decreased compared to the pristine WO_3_ film. The estimated grain size for pristine WO_3_ and Zn: WO_3_ films were around 98 nm and 62 nm, respectively. A smaller grain size facilitated the chemisorption phenomena and the sensitivity of sensors by diffusing the gas throughout the grains and reducing the barrier for charge transfer between the adjacent grains [32].

### 3.4. PL Studies

**Figure 4:**
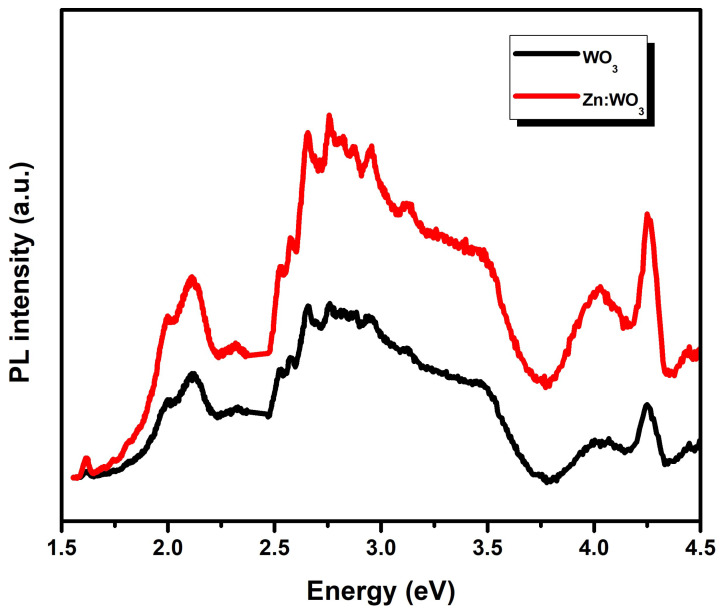
PL spectra of WO_3_ and Zn: WO_3_ films.

PL measurements of pristine WO_3_ and Zn: WO_3_ films were performed at room temperature via JASCO FP 8300 spectrofluorometer excited at 256 nm using a Xe lamp source, as shown in Figure 4. Luminescent centers at 4.25 eV and 4.02 eV correspond to NUV emissions predominantly, which arise owing to the deep level of oxygen vacancies present in the conduction band of the films [33]. A PL emission at 3.47 eV was due to band-to-band transitions. In contrast, the emission center at 2.75 eV is probably due to the interstitial oxygen or other impurities present in the films [34]. The lower energy emission center at 2.12 eV can be ascribed to the localized defects lying within the bandgap of the films [35]. The enhanced emission intensity for Zn: WO_3_ films signified an increased radiative defect density upon Zn doping. The surface states generally increased as the grain size decreased due to a large surface-to-volume ratio. In addition, PL intensity increased as the density of the surface states increased. Thus, films with smaller grain sizes show higher luminescence than larger ones. The results observed are consistent with SEM analysis.

### 3.5. XPS Studies

**Figure 5:**
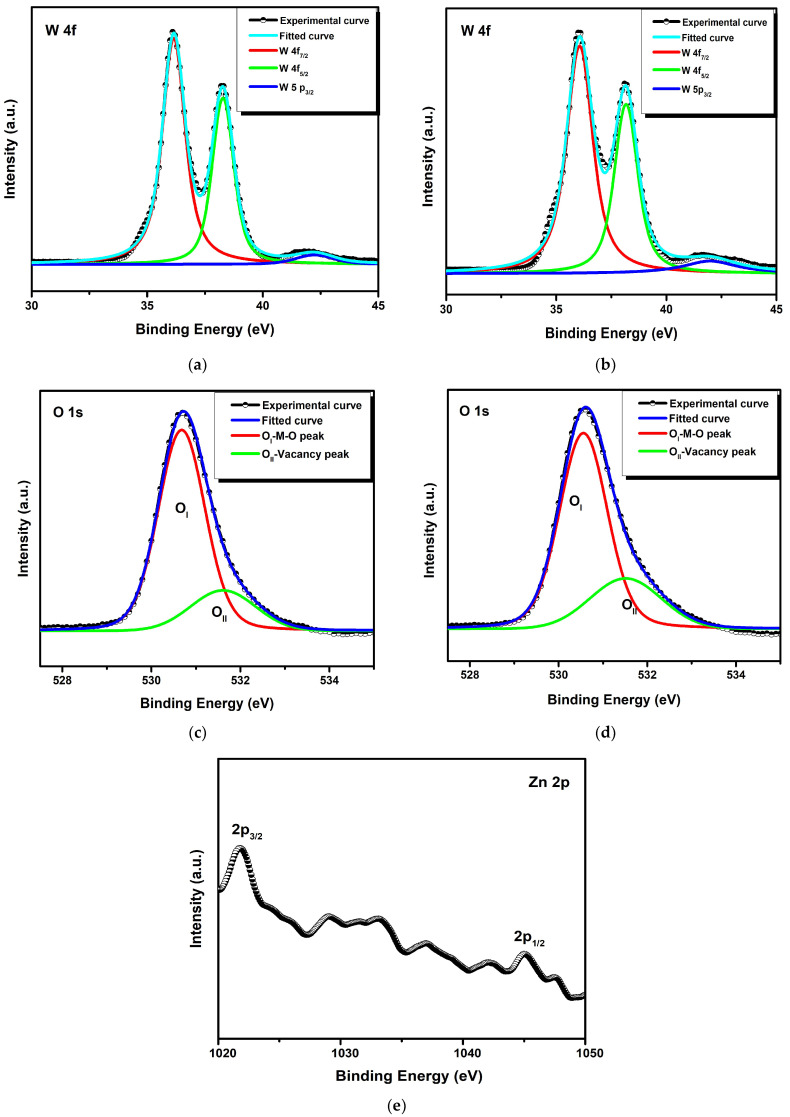
Deconvoluted core level XPS spectra of (**a**,**b**) W 4f (**c**,**d**) O 1s (**e**) Zn 2p in WO_3_ and Zn: WO_3_, respectively.

Chemical state and composition studies were performed using a Kratos AXIS ULTRA X-ray Photoelectron spectroscope. Figure 5a–e represents the deconvoluted XPS spectra composed of W 4f, O 1s and Zn 2p core levels in WO_3_ and Zn: WO_3_ films. The high resolution W 4f peaks (Figure 5a) signifies the W4f_7/2_ and W4f_5/2_ spin-orbit doublet at the binding energies (E_b_) 36.15 eV and 38.28 eV, respectively, corresponding to the +6 oxidation state [11]. Upon Zn dopng, W 4f energy levels shifted (~0.1 eV) toward lower E_b,_ suggesting the emission of electrons from lower oxidation states of W (substoichiometric WO_3−x_) [36]. A satellite peak at 42.24 eV was attributed to W 5p_3/2_. Oxygen 1s spectra (Figure 5c) contained two peaks, namely O_I_ and O_II_ at 530.68 eV and 531.61 eV, respectively, the former of which is associated with the lattice oxygen ($O^{2-}$) bonded to tungsten ions, and latter corresponded to oxygen vacancy (O^−^) formed in the lattice [37]. The slight shift (~0.09 eV) of these peaks towards lower E_b_ was observed in the case of Zn: WO_3,_ which could be attributed to the formation of substoichiometric WO_3−x_. Oxygen vacancies in the films served as better adsorption centres for the gas molecules and contributed to enhancement in the sensing performance [37]. Figure 5e depicts the core level spectra of Zn 2p at the binding energies 1021.84 eV (Zn 2p_3/2_) and 1045.06 eV (Zn 2p_1/2_) with respect to the +2 oxidation state, which confirmed the successful doping of Zn into WO_3_ [6,7].

### 3.6. NH_3_ Gas Sensing Analysis

**Figure 6:**
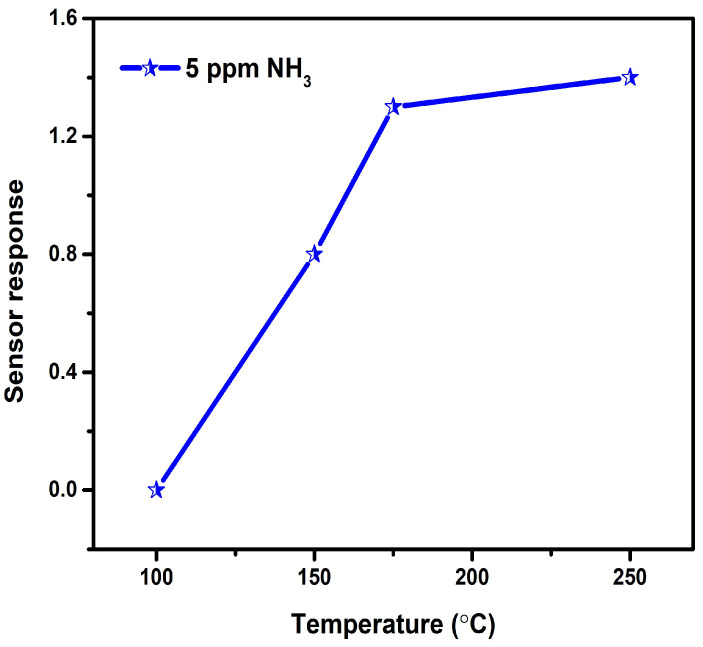
Temperature optimization plot of Zn: WO_3_.

**Figure 7:**
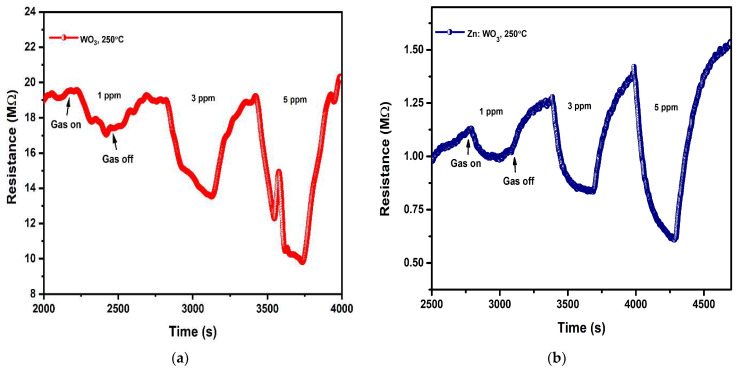
(**a**) Transient response curves of (**a**) WO_3_ and (**b**) Zn: WO_3_ films.

Pristine WO_3_ and Zn: WO_3_ films were exposed to air and ammonia atmosphere at an optimized operating temperature of 250 °C. The temperature optimization plot is given in Figure 6. Transient response curves were recorded for ammonia concentrations of 1, 3, and 5 ppm, as depicted in Figure 7a,b. The sensor response for reducing gases such as NH_3_ is defined by S = ($\frac{R_{a}{-R}_{g}}{R_{g}}$ ) [38,39]. R_a_ and R_g_ are resistances of the film when subjected to air and ammonia, respectively. Response and recovery times are described with a 90% resistance change in the film when it was kept in ammonia and the air, respectively. The sensor response, response time, and recovery time of the films were calculated and reported in Table 2. The variation in the rate of response/recovery is attributed to the change in surface reactions such as the adsorption, diffusion of gas molecules, etc. A higher sensor response of 1.40 was observed for the Zn: WO_3_ film with a response time of 167 s and recovery time of 199 s at 5 ppm NH_3_ concentration. Moreover, Zn: WO_3_ exhibited good sensor performance at concentrations as low as 1 ppm. A smaller grain size observed from SEM increased the surface states observed in PL, and the oxygen vacancy formation noticed from XPS perhaps induced the increment in the response of the Zn: WO_3_ film. Table 3 depicts the comparison study of our work with the previously reported ammonia sensors. The present sensor stands out among the reported literature due to its better response, low detection limit, and moderate operating temperature.

**Figure 8:**
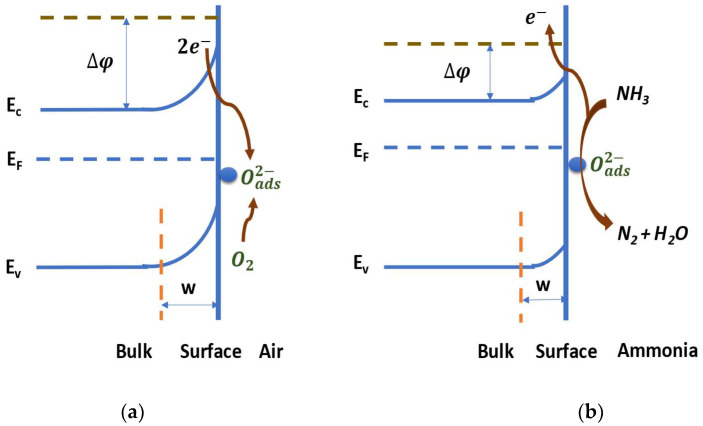
Mechanism of sensing in the presence of (**a**) Air (**b**) Ammonia for Zn:WO_3_ film.

Figure 8 demonstrates the sensing mechanism of metal oxides in the presence of air and ammonia gas. When sensing material (i.e., Zn: WO_3_) is kept in an air atmosphere at a specific temperature, oxygen molecules adsorb on the surface and form ionic species ($O_{2}^{-}{,O}^{-}and\;O^{2-})$ by removing electrons from the conduction band of the film. As a result, the formation of a space-charge layer (w) within the grain and potential barrier (∆φ) and between the grains increases the film’s resistance. When ammonia interacts with the sensor material, the release of electrons to the conduction band occurs through a reduction in the width of ‘w’ and ′∆φ′ and decreases the film’s resistance. The following equations represent the reactions of chemisorbed oxygen species at lower temperatures and their interaction with ammonia [11].

(1) $$O_{2\left(ads\right)}+\;e^{-}\to \;O_{2}^{-}$$

(2) $$O_{2}^{-}+e^{-}\;\to \;2O^{-}$$

(3) $$4{\mathrm{NH}}_{3}(g)+5O_{2}^{-}\to 4NO+6H_{2}O+5e^{-}$$

2NH_3_(g) + 3O^−^ → N_2_ + 3H_2_O + 3e^−^ (4)

**Figure 9:**
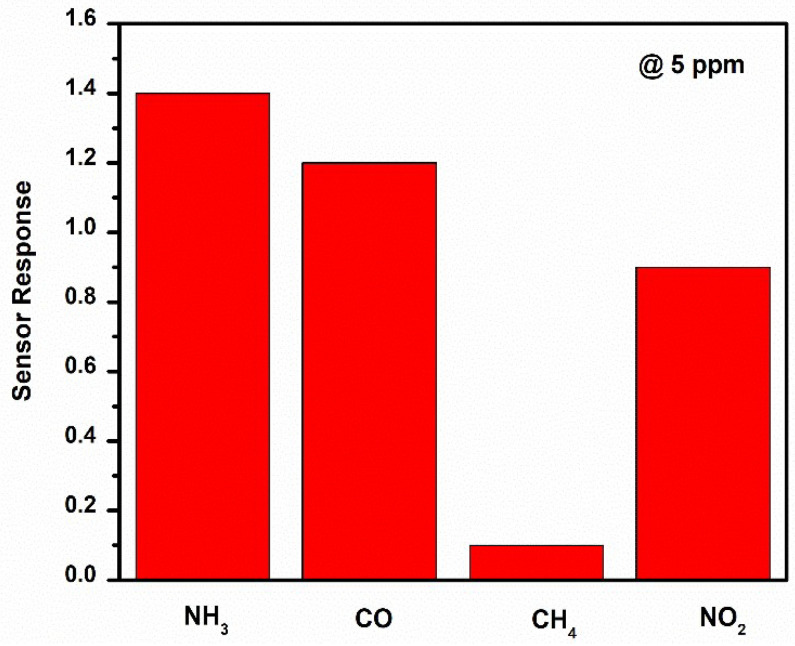
Selectivity studies of Zn: WO_3_ toward various gases.

**Figure 10:**
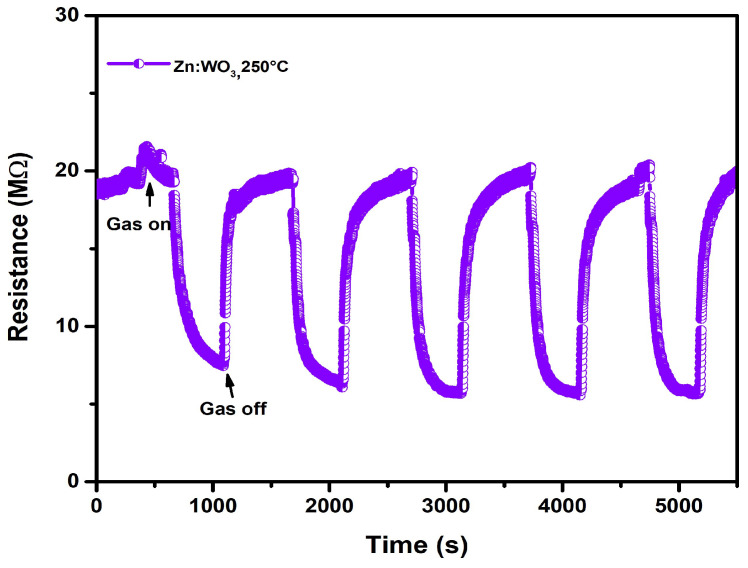
Repeatability measurements of Zn: WO_3_ film.

Selectivity and repeatability are the key aspects that govern the sensing performance of the films. Figure 9 shows the bar graph representing the selectivity of the Zn: WO_3_ film at 5 ppm ammonia concentration. The Zn: WO_3_ film showed a higher response to ammonia compared to the other tested gases, such as CO, NO_2,_ and CH_4_. Figure 10 depicts the repeatable signals of the Zn: WO_3_ film at 5 ppm NH_3_ concentration. The repeatability test conducted for 5 cycles of ammonia exhibited a steady response after each cycle, signifying the excellent repeatability characteristics of the Zn: WO3 film. Since the repeatability measurements were carried out after 8 months due to atmospheric conditions, a change in the resistance was noted. However, we did not observe a large variation in the sensor response of the films.

## 4. Conclusions

Spray-deposited pristine WO_3_ and Zn: WO_3_ films were investigated for their structural, morphological, optical, and ammonia-sensing properties. XRD and Raman confirmed the monoclinic structure of the deposited films. Smaller grains were observed from SEM and oxygen vacancy formation, which were confirmed via XPS and might have ameliorated the sensor response in the Zn: WO_3_ film. Repeatability measurements revealed the good reproducible characteristics of the Zn: WO_3_ sensor toward ammonia gas. Hence, our study confirmed that WO_3_ doped with Zn was a potential approach to enhance the ammonia sensing characteristics at a moderate operating temperature of 250 °C.

## Figures and Tables

**Table 1:** Structural parameters of the films extracted from XRD patterns.

Samples	2θ, (200) (deg.)	FWHM (deg.)	Interplanar Spacing d (Å)	Crystallite Size D (nm)	Dislocation Density δ (×10^14^ Lines/m^2^)	Microstrain ε (×10^−3^)
WO_3_	24.18	0.25297	3.677	32	9.7	1.1
Zn: WO_3_	24.18	0.27097	3.676	30	11.1	1.2

**Table 2:** Sensing parameters of WO_3_ and Zn: WO_3_ films.

	Conc. (ppm)	Sensor Response	Response Time (s)	Recovery Time (s)
WO_3_	1	0.15	156	200
	3	0.40	198	159
	5	0.93	164	123
Zn: WO_3_	1	0.24	89	188
	3	0.65	118	176
	5	1.40	167	199

**Table 3:** Comparison study with the previously reported NH_3_ sensors.

Material	NH_3_ Conc. (ppm)	Sensor Response	Operating Temperature (°C)	Method	Ref.
WO_3_ nanowires	1500	9.7	250	Sputtering and calcination	[40]
WO_3_-Fe_2_O_3_ composites	300	6	300	Hydrothermal	[19]
Pd-WO_3_ films	10	0.27	225	Spray Pyrolysis	[41]
rGO/WO_3_nanowire composites	100	11	300	Hydrothermal	[17]
Ru loaded WO_3_ nanosheets	20	17.8	300	Acidification with impregnation	[42]
WO_3_@SnO_2_ core shell nanostructures	15	1.5	200	Hydrothermal	[43]
Zn: WO_3_ nanostructures	5	1.40	250	Spray Pyrolysis	This work

## Data Availability

The data presented in this study are available on request from the corresponding author.
